# Avoidable Mortality: The Core of the Global Cancer Divide

**DOI:** 10.1200/JGO.17.00190

**Published:** 2018-08-10

**Authors:** Felicia Marie Knaul, Hector Arreola-Ornelas, Natalia M. Rodriguez, Oscar Méndez-Carniado, Xiaoxiao Jiang Kwete, Esteban Puentes-Rosas, Afsan Bhadelia

**Affiliations:** **Felicia Marie Knaul**, Sylvester Comprehensive Cancer Center at the University of Miami; **Felicia Marie Knaul** and **Natalia M. Rodriguez**, University of Miami Institute for Advanced Study of the Americas; **Hector Arreola-Ornelas, Afsan Bhadelia,** and **Xiaoxiao Jiang Kwete**, University of Miami, Miami, FL; **Felicia Marie Knaul**, **Hector Arreola-Ornelas**, and **Oscar Méndez-Carniado**, Fundación Mexicana para la Salud, A.C.; **Felicia Marie Knaul** and **Hector Arreola-Ornelas**, Tómateloa a Pecho A.C., Mexico City; and **Esteban Puentes-Rosas**, Sanofi Pasteur, LATAM Region, Mexico.

## Abstract

**Purpose:**

The incidence of infection-associated cancers and lethality of cancers amenable to treatment are closely correlated with the income of countries. We analyzed a core part of this global cancer divide—the distribution of premature mortality across country income groups and cancers—applying novel approaches to measure avoidable mortality and identify priorities for public policy.

**Methods:**

We analyzed avoidable cancer mortality using set lower- and upper-bound age limits of 65 and 75 years (empirical approach), applying cancer-specific and country income group–specific ages of death (feasibility approach), and applying cancer-specific ages of death of high-income countries to all low- and middle-income countries (LMICs; social justice approach). We applied these methods to 2015 mortality data on 16 cancers for which prevention is possible and/or treatment is likely to result in cure or significant increase in life expectancy.

**Results:**

At least 30% and as much as 50% of cancer deaths are premature, corresponding to between 2.6 and 4.3 million deaths each year, and 70% to 80% are concentrated in LMICs. Using the feasibility approach, 36% of cancer deaths are avoidable; with the social justice approach, 45% of cancer deaths are avoidable. Five cancer types—breast, colorectal, lung, liver, and stomach—account for almost 75% of avoidable cancer deaths in LMICs and worldwide.

**Conclusion:**

Each year, millions of premature cancer deaths could be avoided with interventions focused on four priority areas: infection-associated cancers, lifestyle and risk factors, women’s cancers, and children’s cancers. Our analysis of the global burden and the specific cancer types associated with avoidable cancer mortality suggests significant opportunities for health systems to redress the inequity of the global cancer divide.

## INTRODUCTION

Suffering and death as a result of cancer disproportionately affect the poor. This glaring, unacceptable difference between wealthy and poorer countries is the cancer divide. Shrouded in the myth that cancer is a disease of the wealthy, the divide remains understudied, underprioritized, and undertreated.^1^ Of the 8.7 million global cancer deaths in 2015, more than two-thirds occurred in low- and middle-income countries (LMICs).^2^ Lethality, approximated by mortality/incidence in any given year, is highest in low-income countries for almost all cancers that are screening-detectable or treatable.^1,3^ Although the incidence of infection-associated cancers is inversely associated with income, the opportunity to survive cancers that are amenable to treatment is closely correlated with the income of a country. In 2012, a child living in one of the 25 poorest countries of the world diagnosed with leukemia had approximately a 10% chance of survival, whereas in Canada the figure was close to 90%.^4^

An increasing body of evidence shows that a significant proportion of the cancer burden, especially in LMICs, could be avoided by reducing exposure to risk factors and increasing access to screening for early detection and treatment.^1,3^ However, the necessary public health and medical interventions are much less accessible to the poor, which largely explains the disparate mortality rates between and within population groups.

Our analysis unveils one aspect of the enigma of the cancer divide: avoidable mortality across country income groups. We present estimates of premature cancer mortality using data for 2015^2^ and comparing low, lower-middle, upper-middle, and high-income countries. We apply the concept of avoidable mortality, which refers to premature deaths that, given current medical knowledge and technology, could be avoided by a health system through either prevention and/or treatment and should not occur in the presence of effective and timely health care.^5-7^

We identify 16 types of cancer for which prevention and/or treatment would likely result in a cure or a significant increase in life expectancy and analyze the avoidable mortality associated with each using three distinct conceptual and empirical approaches: specific age cutoffs; assuming that any country should be able to have the same cancer-specific median ages of death as other countries in a group that faces similar economic conditions; and applying the cancer-specific median ages of death of high-income countries to all country income groups, assuming that poorer countries should be able to expect the same outcomes as those of rich countries.

The WHO Global Action Plan for the prevention and control of noncommunicable diseases (NCDs) 2013- 2020, is specifically aimed at reducing premature mortality from NCDs, including cancer, by 25% by 2025.^8^ Our analysis contributes to this global effort by highlighting the large proportion of cancer mortality that should be considered avoidable, measuring the concentration of these deaths in LMICs, and identifying high-priority cancer types that could be targets for health system and other policy interventions and innovations.

## METHODS

Analysis of avoidable mortality assumes a goal for life expectancy of a population and identifies all deaths from a specific cause that occur before that age. The term avoidable mortality originates from efforts to benchmark quality of care through the measurement of unnecessary, untimely deaths.^5^ Initial empirical analysis at the population level was conducted to assess mortality differences in England and Wales and first introduced the terms “avoidable mortality” and ailments “amenable to medical intervention.”^9^^(p691)^

Traditional methodologies, like that of WHO,^10^ use specific age cutoffs, and because of increased global life expectancy, the age at which point a death is no longer avoidable tends to shift upward over time. Until recently, the majority of literature on avoidable mortality had typically established premature death using an age limit of 65 years.^11-15^ As life expectancy increased, the choice of an upper limit of 65 years was no longer justified, and some studies began setting an age limit of 75 years, more closely aligned with life expectancy in developed countries.^16,17^ More innovative approaches include the frontier country methodology that is based on the lowest risk of mortality for each sex-age group across countries.^18^

Our methods build off work by Knaul et al,^1^ which described two additional conceptual approaches beyond that of a 65-year empirical cutoff: a feasibility approach, which used the median age of death in the best-performing country of each income group (low, lower-middle, upper-middle, and high) for each cancer as the threshold; and a social justice approach that used an empirical cutoff of 75 years, which is close to the mean age of death observed in high-income countries, and considers that poor countries should hold the same standard as rich countries. Here, we refine and expand on these conceptual approaches to analyze avoidable mortality associated with 16 cancer types across country income groups, using 2015 Institute for Health Metrics and Evaluation cancer mortality data^2^ and 2015 World Bank country income classifications.^19^

Our selection of cancer types is based on earlier research,^6,11-15^ focusing specifically on those for which prevention and/or treatment would likely result in a cure or a significant increase in life expectancy. The cancer types considered in our analysis are: bladder, breast, cervix-uteri, colorectal, corpus uteri (endometrial), Hodgkin lymphoma, leukemia (in children), lip and oral cavity (including larynx), liver, lung (including trachea and bronchus), non-Hodgkin lymphoma, prostate, skin melanoma (malignant), stomach, testicular, and thyroid.

For a cancer death to be considered avoidable it must have occurred prematurely, as defined in Equation 1:

$$AM_{ij}=\;\sum_{k=0}^{n}D_{k}\;$$ (1)

where *AM_ij_* is the avoidable mortality associated with cancer type *i* in country *j*; *D_k_* is the number of deaths in age stratum k; and *n*, for each scenario, is the age limit from which a death is considered potentially avoidable with appropriate prevention and treatment interventions in place. We applied three different conceptual approaches to establish the age limit below which a cancer death could be considered avoidable.

### Empirical Approach

Under this scenario, a death is considered avoidable if the age at which the death occurred is younger than 65 years, a lower-bound threshold on life expectancy and a minimum cutoff for countries to achieve. An exception to this rule is death as a result of leukemia, for which the age limit in the literature is usually 45 years.^20-22^ This approach follows the majority of the literature and also happens to be the approximate global median age of death in our data across the 15 cancers (all excluding leukemia) (Table 1).^11-15^ In this scenario, n = 64 years in Equation 1.

**Table 1:**
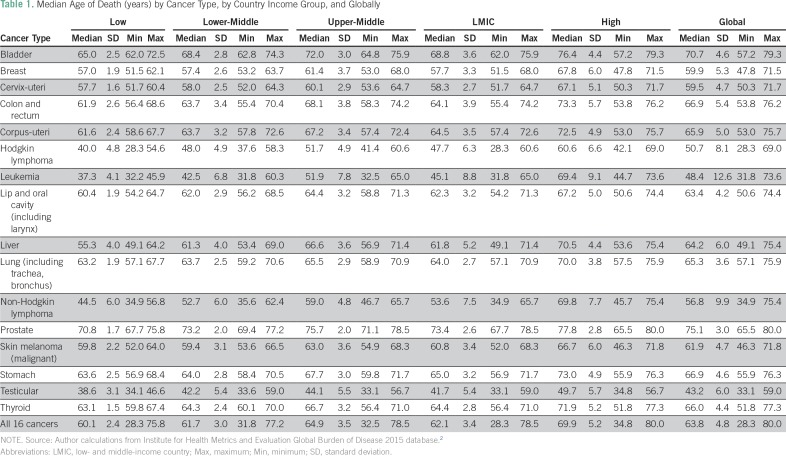
Median Age of Death (years) by Cancer Type, by Country Income Group, and Globally

The empirical approach considers an age limit of 75 years as an upper-bound threshold that is close to overall life expectancy levels observed in high-income countries. Although some top-performing countries have average life expectancies longer than 75 years, disaggregated cancer mortality data above age 75 years are largely unavailable, and this cutoff has also been used widely in the literature.^2,16,17^ In this scenario, n = 74 years in Equation 1.

### Feasibility Approach

The feasibility approach goes beyond the concept of a singular cutoff and applies the median age of death for each of the cancers in each of the four country income groups as the threshold for what can be achieved, thereby establishing 64 separate cancer- and income group–specific age limits (Table 1). This approach assumes that any country should be able to do as well as other countries in a group that faces similar economic challenges and restrictions.

Thus, Equation 1 becomes modified as follows (Equation 2):

$$AM_{ijl}=\;\;\sum_{k=0}^{n_{l}}D_{k}$$ (2)

where *l* corresponds to the income group and takes the values of low, lower-middle, upper-middle, or high. The previous *n* changes to *n_l_* and corresponds to the age limit from which a death is considered potentially avoidable with appropriate prevention and treatment interventions in place in income group *l*.

### Social Justice Approach

The social justice approach applies the median age of death of high-income countries for each of the 16 cancer types as the standard expectancy of age of death to be applied worldwide. This approach focuses on the achievable life expectancy on the basis of the wealthiest countries in the world and reflects the view that residents of poorer countries should be able to expect the same outcomes as those of rich countries and that, on average, people living with cancer in wealthier countries have higher life expectancies. This approach is centered on the principle of fairness, particularly on ensuring equitable health capabilities for all, both the opportunity and process freedoms to achieve better health.^23,24^ In this scenario, *n_l_* in Equation 2 corresponds to the median age of death in high-income countries.

Each of these approaches was applied to each country’s mortality data and age at death by cancer type from the Institute for Health Metrics and Evaluation 2015 Mortality Database.^2^ Total mortality estimates for each cancer type are presented by income group (Table 2).

**Table 2:**
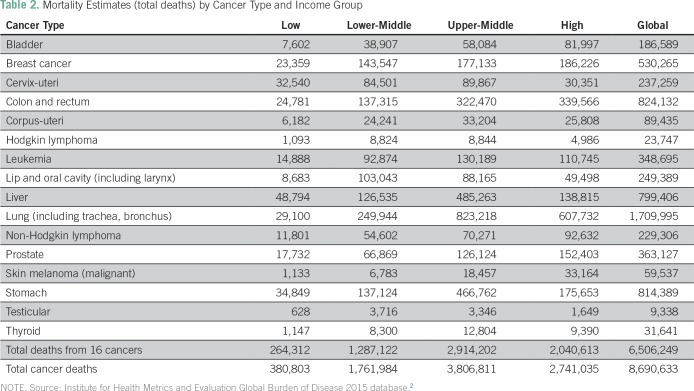
Mortality Estimates (total deaths) by Cancer Type and Income Group

## RESULTS

Using the empirical approach with a threshold of 65 years, approximately 30% (2.6 million) of the 8.7 million global deaths from cancer in 2015 are considered avoidable, of which > 80% occurred in LMICs. Using the most demanding threshold of 75 years, approximately half (4.3 million) of the 8.7 million global deaths from cancer in 2015 could be avoided, > 76% of which occurred in LMICs (Table 3).

**Table 3:**
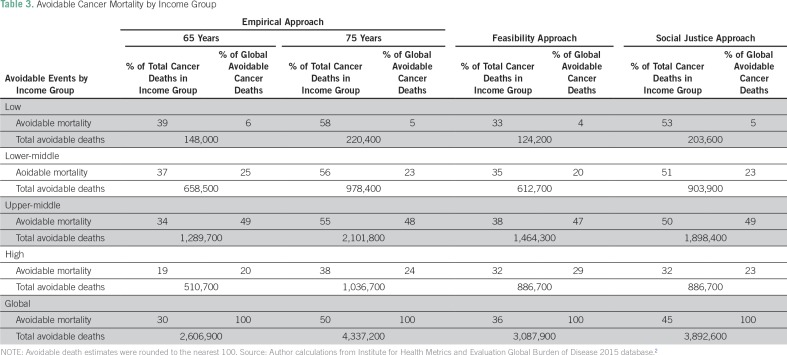
Avoidable Cancer Mortality by Income Group

Applying the feasibility approach, approximately 36% (almost 3.1 million) of the 8.7 million global cancer deaths in 2015 are considered avoidable. The burden of avoidable cancer mortality still falls largely on the poor, as > 71% of global avoidable cancer deaths occurred in LMICs.

Results from the social justice approach suggest that in an idealized scenario, where residents of poor countries are able to expect the same outcomes as those of rich countries currently, up to 45% of all global cancer deaths could be avoided, and > 77% occur in LMICs. This means that nearly 3.9 million deaths could be avoided if the highest standards of medical interventions, programs, and known efficient health policies were made available to all populations at a global level.

We next consider cancer-specific global avoidable mortality as a proportion of total deaths from each specific cancer type (Table 4**)**. Under both the feasibility approach and social justice approach scenarios, the five cancer types with the highest proportions of avoidable mortality globally are testicular, liver, corpus-uteri, cervix-uteri, and breast. Irrespective of the approach used, breast, cervix-uteri, liver, and testicular cancers rank among the top five.

**Table 4:**
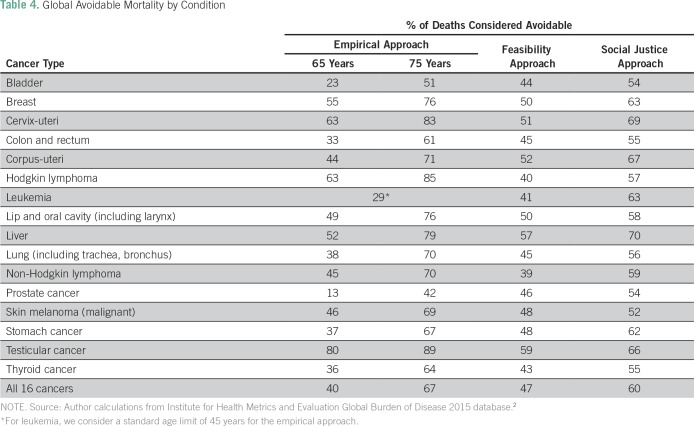
Global Avoidable Mortality by Condition

Overall, the empirical approach lower and upper bounds of 65 and 75 years suggest that between 40% and 67% of global deaths from these 16 cancer types are avoidable. Under the social justice approach scenario, 58% of all global deaths from these 16 cancers are considered avoidable. Considering the economic context and restrictions of each income group separately, the feasibility approach, which we consider the most realistic scenario for all countries, still suggests that nearly 47% of global deaths from these 16 cancer types are avoidable.

We use this feasibility approach to show cancer-specific avoidable mortality by income group and globally (Table 5). Globally, lung cancer is the leading cause of avoidable mortality, accounting for one in four avoidable cancer deaths. Five cancers (lung, liver, stomach, colorectal, and breast) account for almost 75% of all avoidable cancer deaths in LMICs overall and worldwide.

**Table 5:**
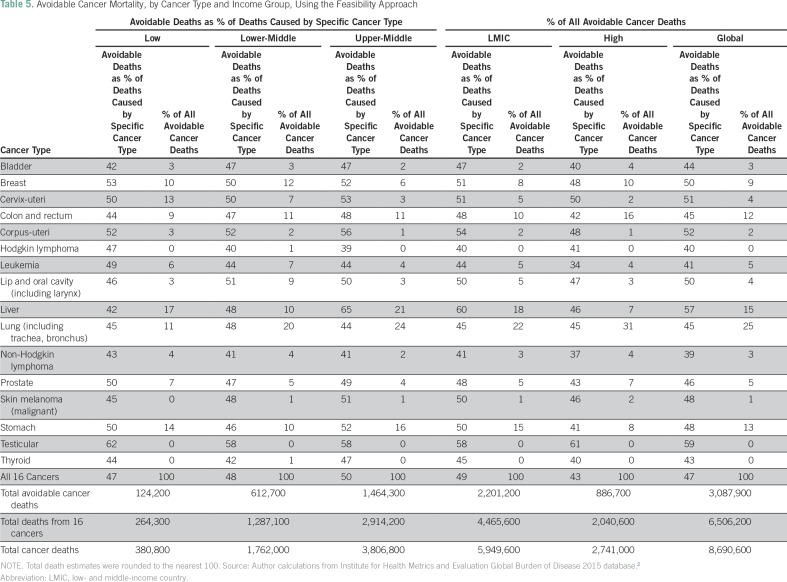
Avoidable Cancer Mortality, by Cancer Type and Income Group, Using the Feasibility Approach

## DISCUSSION

Building off previous work,^1^ we used three different conceptual approaches for estimating avoidable mortality and applied these to a subset of cancers that can be prevented or treated successfully in low-resource settings using current knowledge and medical advances. Our estimates suggest that there are between 2.6 and 4.3 million deaths from cancer each year that could be avoided with effective prevention and treatment. LMICs account for the majority (70% to 80%) of this avoidable cancer mortality.

Using both the empirical and social justice approaches results in a clear gradient from low- to high-income countries in the proportion of cancer deaths that can be considered avoidable. A much larger proportion of deaths in LMICs could be prevented; avoidable mortality as a proportion of total cancer deaths is 1.5 to two times higher in low-income countries compared with high-income countries. Many of these deaths are from infection-associated cancers. Variation is lower using the feasibility approach, and levels of avoidable mortality are uniformly high at 33%, 35%, 38%, and 32% in low, lower-middle, upper-middle, and high-income countries, respectively. This reveals that even in high-income countries, a considerable proportion of deaths from cancer could potentially be avoided with universal access to prevention and/or treatment.

Even with severe resource constraints, a well-conceived and well-managed national cancer program, spanning the cancer continuum from prevention to treatment to palliation, can reduce avoidable cancer mortality and improve the lives of patients with cancer. Understanding the causes and determinants of avoidable cancer mortality has important implications for targeting health policy to those cancers with particularly high avoidable mortality rates and for which cancer-specific interventions should be promoted and prioritized in health financing and social insurance. The evidence can assist countries in identifying both cancers that affect large proportions of the population (eg, lung) and others that affect smaller numbers of people but are highly treatable or preventable (eg, testicular). In some countries, health reform and universal health coverage initiatives have been designed using evidence on avoidable cancer mortality balanced with concerns for equity and feasibility.^25^^,^

Although income and geography should not determine the probability of dying as a result of a disease, in reality they do. LMICs suffer a larger share of global mortality, as compared with global incidence, for almost all cancers that are screening-detectable or treatable.^1^ As science and innovation uncover new methods for early detection, treatment, and cure, the suffering and death as a result of these cancers becomes increasingly concentrated among the poor.

Our findings reveal four key areas that should be prioritized by LMICs, for which significant opportunities exist for health sector policies, programs, and quality medical interventions. These are: controlling risk factors, infection-associated cancers, women’s cancers, and pediatric and adolescent cancers.

Our results for middle- and high-income countries show highest avoidable mortality proportions for lung, breast, colorectal, liver, and stomach cancers, revealing a persistent need to address behavioral risk factors and expand access to screening and early detection. These interventions coincide with the best buys that have been highlighted by the WHO.^26^

Cancers associated with infection account for almost 25% of all cancers^27,28^ and disproportionately affect poor populations. Our results show that the cancers that comprise the largest proportion of all avoidable cancer deaths in low-income countries are liver, stomach, and cervix-uteri cancers. Access to low-cost interventions for primary prevention through vaccination against human papillomavirus or hepatitis B, as well as increasingly low-cost screening, early detection, and treatment of certain infections, should be expanded as part of universal health coverage, with a focus on poor population groups. These cancers are concentrated among the poor in middle- and high-income countries, and prevention and early detection should be a high, targeted priority.^29^

Our results from both the feasibility and social justice approaches reveal the five cancer types with the highest proportions of avoidable mortality globally, in rank order: testicular, liver, corpus-uteri, cervix-uteri, and breast. Three of these five cancers are almost exclusively endured by women, a reality that exacerbates sex inequality worldwide. Furthermore, these cancers tend to affect young women in their reproductive and productive years, accounting for a large number of healthy years of life lost and affecting entire families.^30-32^ This presents a key opportunity for LMICs to integrate early detection programs for women’s cancers into existing health and development initiatives, including maternal and child health, sexual and reproductive health, HIV/AIDS programs, and antipoverty platforms.^1^

LMICs stand to benefit significantly from policies that address childhood and adolescent cancers, like leukemia. These cancers account for a high number of potential years of healthy life lost and are important targets for national cancer planning. Furthermore, the cancer divide is most pronounced for children, with high mortality-to-incidence ratios in the poorest countries of the world and low ratios in the United States, Canada, and Western Europe due to access to treatment.

Our research has several limitations that point to opportunities for future work. Our calculations provide point-in-time estimates of avoidable mortality for 2015 but do not speak to future trends. Although our estimates are likely to effectively capture the potential to reduce the impact of infection-associated cancers for which rates of incidence and mortality are high in lower-income countries and low in high-income countries (eg, cervical cancer), they will likely understate the potential to reduce the burden of cancers that are currently of low incidence in LMICs. For diseases such as breast cancer, where prevention is difficult and for which incidence is expected to increase as countries undergo epidemiologic and demographic transitions, our methodology will underestimate the associated avoidable mortality. Additional research should be undertaken to project the stream of future avoidable mortality, accounting for these transitions. In addition, in countries with a high burden of HIV and cancer comorbidity, the cancer-specific age at death would be lower, and future analysis should consider comorbidities. Furthermore, our estimates are based on overall mortality estimates for all ages, and future research should replicate the analysis specifically for children and by sex. Results should also be disaggregated by geographic region, and country-specific results should be analyzed where national cancer registries exist to identify country-specific priorities.

Each year millions of premature cancer deaths could be avoided with low-cost interventions focused on reducing risk factors, promoting screening and early detection, and providing access to basic treatment. Despite vast inequities in access to cancer medicines, vaccines, and technologies, effective low-cost treatment, financial protection programs, and innovative care delivery models exist and can be applied to resource-constrained settings to reduce avoidable cancer mortality worldwide.^1,33,34^ The results presented herein can facilitate the design of national cancer control plans and strategies to expand access that should be rooted in a comprehensive care continuum from public health prevention strategies, to screening and early detection interventions linked to accessible and quality treatment, to palliative care required to relieve the pain and suffering associated with all cancers.^1,35^
